# Long-Term Use of a Static Hand-Wrist Orthosis in Chronic Stroke Patients: A Pilot Study

**DOI:** 10.1155/2013/546093

**Published:** 2013-02-27

**Authors:** Aukje Andringa, Ingrid van de Port, Jan-Willem Meijer

**Affiliations:** Revant Rehabilitation Centre Breda, Brabantlaan 1, 4817 JW Breda, The Netherlands

## Abstract

*Background*. Long-term splinting, using static orthoses to prevent contractures, is widely accepted in stroke patients with paresis of the upper limb. A number of stroke patients complain about increased pain and spasticity, which leads to the nonuse of the orthosis and a risk of developing a clenched fist. *Objectives*. Evaluating long-term use of static hand-wrist orthoses and experienced comfort in chronic stroke patients. *Methods*. Eleven stroke patients who were advised to use a static orthosis for at least one year ago were included. Semistructured telephone interviews were conducted to explore the long-term use and experienced comfort with the orthosis. Data were analyzed using descriptive statistics. *Results*. After at least one year, seven patients still wore the orthosis for the prescribed hours per day. Two patients were unable to wear the orthosis 8 hours per day, due to poor comfort. Two patients stopped using the orthosis because of an increase in spasticity or pain. *Conclusions*. These pilot data suggest that a number of stroke patients cannot tolerate a static orthosis over a long-term period because of discomfort. Without appropriate treatment opportunities, these patients will remain at risk of developing a clenched fist and will experience problems with daily activities and hygiene maintenance.

## 1. Introduction

Of all stroke survivors, more than half experience impairments of the upper limb in the chronic phase, including loss of strength and dexterity, spasticity, muscle contracture, pain, and edema [1–3]. Patients with a more severe paresis have a higher risk of developing spasticity [4] and muscle contractures of the wrist and finger flexor muscles [5–7]. Without appropriate spasticity treatment or contracture prevention, patients are at risk of developing a clenched fist, a hand which is deformed into a fist by shortening of flexor muscles of the fingers and soft tissue [8]. The abnormal position of the hemiplegic hand and wrist due to spasticity and muscle contractures may interfere with daily activities and hygiene maintenance, both negatively influencing the quality of life [9–11].

Different approaches are used to inhibit spasticity, prevent contractures, reduce pain and edema, or improve hygiene maintenance of the hand in stroke patients with a nonfunctional spastic upper limb. However, there is no consensus about the most effective treatment [12]. A commonly used and widely accepted intervention is prolonged splinting using static orthoses [12–17]. Two reviews on the effect of upper limb splinting after stroke have been published [18, 19]. Both reviews showed no effect of static orthoses on upper limb function, range of motion, and pain after an intervention period less than 13 weeks. However, conclusions should be interpreted with caution because of the lack of high quality randomised controlled trials. There is a considerable heterogeneity of included study designs, clinical aims, and orthosis wearing protocols, materials, and regimes. In addition, all published studies focused on the short-term effect of splinting with splinting periods no longer than 13 weeks. Despite controversies concerning splinting of the hemiplegic upper limb, static orthoses continue to be advised in clinical practice.

When used in clinical practice, a considerable amount of stroke patients complain about increased pain and spasticity since the use of the static orthosis [20, 21]. Due to discomfort, the orthosis cannot be worn for the advised 8 hours per day which leads to nonuse in chronic stroke patients and with that increases the risk of developing clenched fists with which patients may experience problems with daily activities and hygiene maintenance.

Given our experiences in clinical practice, the purpose of this pilot study is to describe the long-term use of static hand-wrist orthoses and the experienced comfort of wearing the orthosis in chronic stroke patients in order to acquire preliminary data to further study the treatment of this specific patient population. We hypothesize that, in a number of the chronic stroke patients with upper limb impairments, discomfort—increased pain, and spasticity—is the reason for not wearing a static hand-wrist orthosis for the advised 8 hours per day. The secondary aim is to describe the self-reported complaints before and since the use of the static orthosis to evaluate the effect of the use of the orthosis in chronic stroke patients. Additionally, the use of cointerventions for the impaired upper limb is investigated.

## 2. Methods

**Figure 1:**
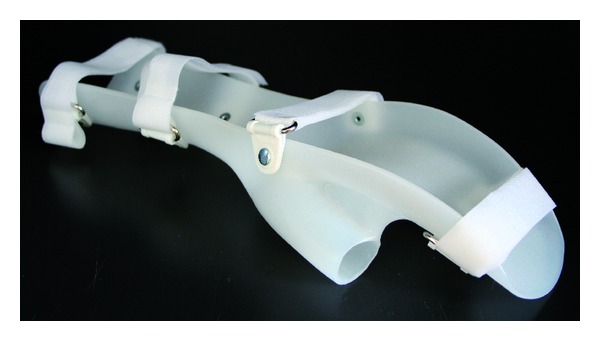
Example of a prefabricated static hand-wrist orthosis.

In this pilot study, semistructured interviews were used to explore the long-term use (i.e., more than one year) of the static orthosis in chronic stroke patients, and the experienced comfort with the static orthosis in chronic stroke patients (Figure 1). A selection of stroke patients, who received a static orthosis from the Orthopaedic Centre OIM Brabant Breda, The Netherlands, was taken from the database. All stroke patients who were advised to use a static orthosis at least one year ago and were independently living in the community were included. Patients were excluded when correct contact details were missing or when patients died in the study period. If patients were unable to communicate by telephone, information was obtained from the primary caregiver. Informed consent was obtained prior to each interview. 

Patients were asked about current use, comfort of the orthosis, reasons for wearing the orthosis, self-reported complaints in the hemiplegic upper limb, including spasticity, hygiene maintenance, pain, and edema, and applied cointerventions. Answers to all twelve questions were scored categorically except the complaints scores. Complaints scores were graded from 0 (no complaints) to 10 (major complaints). The telephone interviews were carried out by a physical therapist who was not directly involved in the patients' treatment. 

Descriptive statistics were used to analyze the results of the semistructured telephone interviews. Nonparametric analyses were applied to evaluate self-reported effect of the orthosis by comparing the data of complaints before and since the use of the static orthosis using a Wilcoxon's signed rank test. Statistical analysis was performed using SPSS 18.0. Statistical significance was set at the 5% level.

## 3. Results

### 3.1. Study Population

A total of 38 patients, diagnosed with stroke, received a static hand-wrist orthosis at the Orthopaedic Centre OIM Brabant Breda, The Netherlands, between January 1, 2008 to October 1, 2009. Participants were retrospectively recruited from the database at October 1, 2010. Nineteen stroke patients matched our inclusion criteria and were invited for the study. Eight patients could not be interviewed, since three died between receiving the static orthosis and data collection, and five could not be reached by telephone. Data of eleven patients (7 female, 4 male, median age 54 years, range 23–80 years) was collected. One interview was conducted with a caregiver. All patients were in chronic stage after stroke (median 86 months poststroke, range 27–163 months) and were advised to use the static orthosis for at least one year ago. 

### 3.2. Long-Term Use of the Orthosis

As shown in Table 1, after at least one year from receiving the static orthosis, three patients still wear the orthosis during night time, with different experienced comfort. Four patients wore the orthosis for at least 8 hours per day, all with good experienced comfort. Two patients were unable to wear the orthosis prescribed 8 hours per day, due to poor comfort. Two patients stopped using the orthosis, one because of an increase of spasticity and the other because of an increase in pain.

### 3.3. Self-Reported Complaints in the Hemiplegic Upper Limb

The main reasons reported for wearing the orthosis were reducing spasticity (10/11), improving opening of the hand (7/11), and improving hygiene maintenance of the hand (5/11) (see Table 2). None of the patients wore the orthosis to reduce edema. The complaints score since the use of the orthosis showed a decreasing trend; however differences between complaints in the hemiplegic upper limb before and since the use of the orthosis were not significant (*P* < 0.05).

### 3.4. Cointerventions

Ten patients reported cointerventions for their upper limb impairments of whom eight were still using the static orthosis (Table 1). Six patients received regular physical therapy sessions, six patients performed daily home exercises, and two patients used spasticity medication (Botulinum toxin or Baclofen). Only one patient did not use any other form of intervention for the impaired upper limb.

## 4. Discussion

In this pilot study, we investigated the long-term use of a static hand-wrist orthosis in chronic stroke patients. Of the 11 interviewed stroke patients, two stopped wearing the orthosis because of discomfort and two could not endure the orthosis for the prescribed wearing time of at least 8 hours per day. These findings support our hypothesis that a substantial number of patients who are at risk of developing contractures in the upper limb, that is, four of the eleven, are not able to endure a static orthosis for the prescribed 8 hours because of discomfort. Concluding that these chronic stroke patients do not receive the appropriate intervention to prevent contractures. Of the seven patients who used the orthosis as prescribed, that is, at least 8 hours, two still complained of discomfort. Without appropriate contracture prevention, these patients will develop contractures in the upper limb which can lead to problems during daily activities and hygiene maintenance, both negatively influencing the quality of life.

The experienced discomfort can be a result of the static characteristics of the orthosis. The position of the orthosis sets the wrist in a fixed position. However, the level of spasticity varies during daytime resulting in different positions of the wrist. With a higher level of spasticity of the muscles, the wrist tends to flex. In contrast, a lower muscle tone can lead to less flexion, or even extension, of the wrist. The chosen position of the static orthosis is seldom adequate to manage these varying levels of spasticity and changing ranges of wrist mobility. When spasticity increases, the hand and fingers will try to flex in the rigid orthosis which causes pain and discomfort. For stroke patients with varying levels of spasticity in the upper limb, a static orthosis with a fixed position of the wrist can lead to problems tolerating the orthosis. 

Taking this into account, an orthosis for the prevention of contractures in the spastic upper limb needs to allow higher levels of spasticity and flexion of the wrist. A dynamic orthosis using the low-load and prolonged stretch principle, with a hinge which allows the wrist to flex during higher levels of spasticity, might be more appropriate for these stroke patients.

In our study, most patients use the orthosis because of spasticity in the upper limb, to prevent contractures or to preserve the ability to open the hand for hygiene maintenance. Patients in our study did not report a significant difference of the complaints concerning spasticity, contracture, or pain before and since the use of the static orthosis, although the complaints tended to decrease in this small sample size group. Previous studies on the short-term effect of the static orthosis indicate that stretch does not have clinically important immediate or short-term effect on joint mobility [22]. There is evidence indicating that static orthoses show no effect on upper limb function, range of motion at the wrist, fingers, or thumb, nor pain [19, 23]. Despite the lack of studies of long-term effect, physicians and patients still believe that splinting is an appropriate intervention for contracture prevention. Because contractures develop slowly, studies about the effect of splinting need to focus on long-term use of at least six months. All previous studies focused on an increased joint mobility of the wrist as an effect of the static orthosis. In our opinion maintaining the range of motion of the wrist is already a positive effect of an intervention aiming to prevent contractures.

In conclusion, a static orthosis can be a useful prevention of contractures for a selection of the stroke patients who can tolerate this low-cost orthosis. However, there is a group of chronic stroke patients which is not able to endure a static orthosis and which needs another intervention for the prevention of contractures in the upper limb. In this group, stepped care can be used; when static orthoses are not endured, another intervention has to be applied, for example, dynamic orthosis. 

### 4.1. Limitations of the Study

Although explorative, this study offers insight into the long-term use of a static orthosis in chronic stroke patients and the patient's experiences with it. Despite the preliminary character of this study, the presented data are the first about long-term use of the static orthosis and experienced comfort. Patients had to recall scores of complaints before the use of the static orthosis which could have been influenced by recall bias. Taking this into account, in combination with the small sample size, the results about the self-reported complaints of static splinting should be handled with care and should be confirmed in larger studies.

### 4.2. Further Research

Further studies are important to identify the stroke patients who are able to tolerate the static orthosis and patients who will need other interventions to prevent the development of contractures. For these specific stroke patients who are not able to tolerate the commonly used static orthosis, it will be relevant to study the effect of alternative interventions, for example, dynamic orthoses.

## 5. Conclusion

These pilot data show that a number of chronic stroke patients cannot tolerate a static orthosis for at least 8 hours per day during a long-term period of at least one year. Without appropriate treatment opportunities, these patients will remain at risk of developing a clenched fist and will experience problems with daily activities and hygiene maintenance. It is, therefore, worthwhile to find other interventions which can be endured by these stroke patients.

## Figures and Tables

**Table 1:** Orthosis wearing time, experienced comfort, and the reported cointerventions in addition to the use of the orthosis.

Patient	Wearing time per 24 hours	Day/night use	Reported comfort	Cointervention
1	4–6 hours	Day	Poor	Handmaster
2	>8 hours	Night	Poor	None
3	0 hours (nonuse)		Very poor	Medication, home exercises
4	>8 hours	Night	Poor	Physical therapy
5	6–8 hours	Day	Very poor	Physical therapy, home exercises
6	>8 hours	Day	Good	Physical therapy, home exercises
7	>8 hours	Day	Good	Home exercises
8	0 hours (nonuse)		Poor	Home exercises
9	>8 hours	Day	Good	Physical therapy
10	>8 hours	Day	Good	Medication, physical therapy, home exercises
11	>8 hours	Night	Good	Physical therapy

**Table 2:** Self-reported complaints in the hemiplegic upper limb.

	Reasons for wearing a static orthosis	Complaints before orthosis use	Complaints since use orthosis
		Median (range) (0–10)	Median (range) (0–10)
Spasticity	10/11	8 (5–10)	7.5 (5–10)
Hygiene maintenance	5/11	7 (5–10)	6 (1–7)
Pain	3/11	8 (7–10)	7 (0–9)
Edema	0/11	—	—
Opening hand	7/11	8.5 (7–10)	7.5 (5–10)
